# Intraoperative Identification of the Intersegmental Plane: From the Beginning to the Future

**DOI:** 10.3389/fsurg.2022.948878

**Published:** 2022-07-08

**Authors:** Xianfei Zhang, Chengqiang Li, Runsen Jin, Hecheng Li

**Affiliations:** Department of Thoracic Surgery, Ruijin Hospital, Shanghai Jiao Tong University School of Medicine, Shanghai, China

**Keywords:** segmentectomy, intersegment plane, inflation–deflation method, indocyanine green, fiberoptic bronchoscopy

## Abstract

Segmentectomy has played a crucial role in the treatment of early-stage lung cancer after the publication of JCOG0802, which indicated that patients with small-sized peripheral non-small-cell lung cancer could receive better survival from segmentectomy than lobectomy despite a higher local recurrence. The intraoperative identification of the intersegmental plane ensures complete resection of the lesion with sufficient margin so that it is deemed as the critical part of segmentectomy. Diverse methods have been developed to acquire distinguishable and lasting borderline between segments, but none of them is proved perfect. In this review, we searched and classified these techniques that emerged from the beginning when segmentectomy was used for bronchiectasis until now. Comparisons between different ways in mechanisms, facility, and safety were made to depict a comprehensive landscape for surgeons to select fit one. Furthermore, we presented our vision for the future of intersegmental plane identification.

## Introduction

Segmentectomy was first applied to treat bronchiectasis (1) and, subsequently, lung cancer patients who cannot tolerate lobectomy. With the publication of the outcomes of JCOG0802, the role of pulmonary segmentectomy in small-sized peripheral non-small-cell lung cancer (NSCLC) was confirmed. Patients with clinical stage IA NSCLC who received segmentectomy had better survival than lobectomy, despite the higher local recurrence rates (2). It seems almost inevitable that a much larger amount of segmentectomy will be adopted in early-stage NSCLC patients.

A definite intersegmental plane is required through surgery to make a precise excision and enough margin to minimize the recurrence rates. Various methods had been developed to identify the intersegmental plane. Here, we reviewed and classified them into two main categories based on the target that was emphasized and summarized their merits and demerits. The comparison between methods aims to assist surgeons select fit ones.

## Highlight the Target Segment

To make the target segment distinct through inflation or staining is a straight thought, as the segment possesses bronchi and vessels by nature. Considering that part will be removed later, theoretically, less harm should be done to the rest tissue.

### Inflate the Target Segment

#### Inflation-Deflation Method

This method was introduced by Edward et al. for lingula segmentectomy in bronchiectasis (1). The pulmonary was ventilated by positive pressure gas at the beginning of the operation. The anesthetist is then instructed to release the positive intratracheal pressure after the dissection of vessels and bronchus of the lingula segment, allowing the other lobe to deflate. At the same time, a light clamp is applied to the bronchus to close the outlet of the target segment that will remain inflated for removal.

Kohn's pores on the intersegment plane may diffuse positive pressure gas, leading to an ambiguous borderline. Tsubota modified this method in 2000. The author identified the target segmental bronchus and then inflated the lobe. The bronchus was tied up to keep gas inside and cut off at the proximal point. Thus, the preserved part could release gas through the cut. The author described that compared to the conventional ways in the textbook, a more distinguishable borderline would appear when the stump closing was almost done in virtue of the disuse of positive pressure ventilation (3).

To ligate the resected bronchus was put forward by Oizumi. A modified Roeder knot was made outside the thorax and then tightened after ventilation of both lungs. The borderline would also emerge when unilateral ventilation was resumed (4). The operation time could be shortened for eligible patients (5).

On the contrary, another way took advantage of gas diffusion by Kohn’s pores. Wang et al. identified and dissected the targeted segment bronchus, artery, and intrasegmental vein by ligation or stapler cutting. Then, the collapsed lung was re-expanded completely with controlled airway pressure under 20 cm H_2_O. The air in the preserved segment would be carried away by blood or released through intubation. Thus, the resected segment would remain inflated after 5–12 min (6).

This method moves forward ligation or excision of target bronchia and vessels ahead of inflation of the whole lobe, which may limit the view and operation of surgeons. Meanwhile, no exceptional materials or equipment are required. Consequently, it is widely accepted by thoracic surgeons and known as the “modified inflation–deflation method” and becomes a base way for newly developed methods to compare. Yao et al. evaluated the success rate of this method and grade the borderline according to the intelligibility and found that up to 95.2% of cases in the complex segmentectomy group and 97.6% in the simple segmentectomy group received a satisfactory demarcation for surgery (7).

However, several limitations accompany the modified inflation–deflation method. First, only precise recognition and dissection of structures, which should be carefully identified before surgery, brings to the correct intersegmental plane. Three-dimensional reconstruction of pulmonary structures is now gradually applied to the preoperative designation for accuracy. Second, the waiting time for the borderline prolongs the surgery time. Yang replaced the pure oxygen (O_2_), the conventional gas for inflation, with mixed nitrous oxide (75% N_2_O with 25% O_2_ or 50% N_2_O with 50% O_2_). The time for intersegmental plane emergence was significantly shorter in the 75% N_2_O group without additional complications, despite a lower level of arterial oxygenation after lung expansion (8). The third limitation exists in patients with chronic obstructive pulmonary diseases (COPDs), who lost their normal pulmonary function of contraction and expansion. The borderline in these patients may remain blurry after a long wait, while no improved technique could be applied.

#### Selective Segmental Jet Injection

Directly delivering the gas to the target segment while maintaining the rest part in the deflation is another way to reveal the borderline. The segment can dilate instantly without any delay. Moreover, the process has no requirement for the normal pulmonary function of contraction.

With the development of anesthesia, double-lumen endobronchial intubation and one-lung ventilation become the conventional technique for thoracic surgery. Matsuoka utilized a fibrobronchoscope (FOB) through a tracheal tube to find out the target bronchus, injected a jet into it and then ligated the bronchus to keep jet stay (9). Okada evaluated the feasibility and safety of this selective jet injection by a high-frequency jet ventilator (HFV) in 52 cases, indicating that a distinguishable borderline could be revealed in all patients without severe complications (10). Taguchi used a manual jet ventilator (MJV) instead of HFV for easy mobility and real-time adjustment for the inflation time as well as the amount of aeration, demonstrating a success rate of 89% in 171 cases (11).

In this method, gas leaks through the gap between FOB and bronchus can lead to an unclear intersegment plane. Shi introduced an endobronchial blocker installed before ventilation of the target segment to prevent gas leaks (12). Furthermore, selective jet injection by FOB has a high demand for equipment, particularly the special ventilator. Therefore, some surgeons are seeking more facile ways. Kamiyoshihara et al. inserted a 23-gauge butterfly needle into the distal part of the segmental bronchus that was controlled by a right-angled clamp. Then, the oxygen flow would be instilled through an extensive tube linked to the needle (13). Jiao successfully identified the intersegmental plane with needle insertion in 85 patients of 96 (14). However, this method takes a risk of aeroembolism, from which one patient died (15), as a result of insertion into the segmental artery by mistake. Drawing back to check the location of the tip of the needle should be done before ventilation (14).

A more secure way is to cut the segmental bronchus and insert a soft tube into the distal part for gas delivery. Soultanis inserted the tip of a central venous catheter, which connected to a 60-ml Luer-slip syringe for pumping gas, through partly cut bronchus (16). Kobayashi compared this open-blow method using a 20G plastic cannula with high-frequency jet ventilation (HFJV) and found that complete segmentectomies were achieved more in the former group (90.2% vs 71.1%, *p* = 0.04) (17).

### Stain the Target Segment

Pulmonary alveoli accommodate air by nature, so they also provide the space for dyes. Dye injection into segmental bronchus is developed for better identification in the deep part of the lung, which is less invisible by inflation methods. Meanwhile, the borderline can be maintained for a longer time. Injection through the target segment artery is rarely adopted because of its difficulty in operation.

#### Indocyanine Green Injection

Indocyanine green (ICG) is a water-soluble fluorophore that can emit light in the background of near-infrared (NIR). Sekine introduced an endobronchial tube to transmit diluted ICG to the target segment and then 200– 300 ml of air. Positive end-expiratory pressure was kept at 5 cm H_2_O during the surgery. A thoracoscope and an ICG fluorescence endoscope were simultaneously applied, and a clearer intersegmental plane was maintained in the view of the latter (18). Wada used virtual bronchoscopy to design a more accurate way to guide ICG injection with a success rate of 87% in 15 patients (19). One of the failed cases arose from insufficient ICG injection, while the other case was caused by an influx of excess ICG to preserved part. Anayama tested and found out that the optimal cutoff volume of dilute ICG was 8.91% of the predicted volume of the target segment (20).

Insertion of an intravenous cannula into the bronchus that has been cut off through a surgical site for ICG delivery was introduced by Oh. The segment turned green without a near-infrared device (21). Funai used an endobronchial tube instead of an intravenous cannula. A special near-infrared fluorescence imaging system, PDE-neo, was implemented in the surgery to acquire a high-contrast borderline (22). These methods are in need of separation of segment vessels beforehand in case the ICG is carried away by the blood flow. The most important is that ICG influx to other preserved segments through bronchia is curbed as the target bronchus is separated before injection.

Generally, ICG is a safe fluorescence instrument not only for surgery but also for the determination of the liver and heart function. However, ICG has the potential to cause allergy (23), which is supposed to be noticed in clinical applications.

#### Methylene Blue Injection

Methylene blue is a dye widely used in surgery. It was utilized as a mark for small pulmonary nodule location, which was guided and confirmed by the preoperative image in early time (24, 25) and by endobronchial ultrasonography (26) or even electromagnetic navigational bronchoscopy (27) nowadays for the sake of further pulmonary resection. The exploitation of methylene blue for intersegmental plane identification was introduced by Zhang, who tested the different routes of injection and illustrated that endobronchial injection was the most facile and safe way (28). Analogously to ICG injection, bronchia and vessels to the resected segment should be separated beforehand. All 14 patients were successfully performed predesigned pulmonary resection in this way.

#### Vitamin B2 Injection

Nutrition that exists in the body by nature was paid a lot of attention to be safer fluorescers. Vitamin B2 is a water-soluble substance that served as a coenzyme of oxidative phosphorylation and can be exited to illuminate by near-ultraviolet light of which the wavelength is approximately 450 nm. Waseda et al. introduced the fluorescence technique of vitamin B2 through bronchus in pig models ex vivo and *in vivo*, indicating that an extremely clear intersegmental plane could be observed in all test samples and remained in high contrast even one hour later (29, 30). Yet, the clinical application of this method has not been carried out.

## Give Prominence to Preserved Part

The blood flow of the target segment will be interrupted after ligation and separation of the relative vessels. Drugs injected into the vein can be transported to the other part that preserved blood circulation and be illuminated by a special camera. The infrared thermography can even directly identify the intersegmental demarcation in terms of the temperature discrepancies without any medication.

### Intravenous Fluorescers Application

#### Intravenous Indocyanine Green Injection

Misaki et al. first set up an animal model and then successfully applied this method to eight patients (31, 32). The artery supplying the target segment was ligated at the beginning. An infrared thoracoscopy system was implemented to monitor the changes after ICG intravenous injection. The stained part of the lung could be seen in 13 s, while the peak was attained in 28 s. The light lasted for about 3.5 min, which was described enough for borderline marking, although the time is sharply shorter than the bronchial injection way. Nevertheless, this way has no need for opening the bronchial route, which may present an outlet for tumor diffusion and saves surgical time.

The method is soon accepted for its simplification of surgical steps and appraised for safety, efficiency, and factors influencing illumination. Similar to Misaki’s report, the color discrepancy arises in 10–25 s and lasts for 90–180 s (33–35). It advised clamping the pulmonary vein temporarily to prolong the effective visible time. They dissected and surrounded the lobe vein with a vessel tape at the hilum, tightened it up after ICG injection, and found out that the borderline could be seen as long as the vein was controlled. The clamping was recommended to be relaxed within 5 min, preventing thrombosis in pulmonary veins (36).

The research on uniformity between the intravenous ICG injection and modified inflation–deflation method was carried out by Sun et al. Two methods were simultaneously applied to the same patient, proving a concordant and matched intersegmental demarcation (37). Xie et al. designed a randomized controlled trial to compare these two methods in two parallel groups, indicating that operation time was substantially curtailed in ICG application without significant differences in perioperative parameters and postoperative pulmonary function (38).

Distinct from inflation methods, ventilation function exerts a faint impact on the efficiency of ICG visuality. Pischik demonstrated that COPD, though with a shorter visible time, did not impair the borderline identification, except for one failure in a patient with severe emphysema (34). Furthermore, a retrospective propensity-score matched analysis in patients with COPD showed that intravenous ICG injection served relatively higher success rate (91.4% versus 64.2%, *p* < 0.01) (39). However, smoking or other conditions that obstruct the penetrability of light like anthracosis could reduce the contrast between resected and preserved parts (40).

In summary, intravenous ICG injection provides an expeditious, facile, and accurate way to identify the intersegmental plane regardless of the patients’ pulmonary function. No ICG-related adverse events were observed in the research mentioned above. Yet, like intrabronchial application, the potential side effect of ICG exists and can be even more severe.

#### Intravenous Vitamin B2 Injection

The team of Waseda then explored the intravenous vitamin B2 illumination in pig models after the intrabronchial route. The intersegmental plane was satisfactory after injection and lasted for a duration of more than one hour, which was significantly longer than that of intravenous ICG illumination. What is more, the borderline revealed by vitamin B2 was completely identical to that revealed by the inflation–deflation method and ICG injection (41). Thus, vitamin B2 might be a promising substitution for ICG, though there are no investigations on human beings.

### Infrared Thermography

Blood transfused by the artery not only carries various substances but also heat. The target segment that loses its blood supply shall cool down after relevant artery ligation. Sakamoto et al. used a thermographic monitor to detect the temperature discrepancy after blood interception and found that the target region cooled down by 1.5 cent degrees and could be monitored in about 3.9 min. Meanwhile, the borderline depicted by the thermograph had a good match to that of the inflation–deflation method (42). Li et al. applied this method to two patients and achieved a distinguishable borderline, which though demonstrated a smaller area than the inflation–deflation method. The particular merits of this method include no need for excess drug administration that may bring side effects and no need for inflation that limits the surgical view. Nevertheless, more clinical research is required to evaluate its accuracy and efficiency.

## Discussion

Segmentectomy has been accepted for small-size early-stage NSCLC patients as favorable evidence accumulated. The accurate identification of the intersegmental plane is of great importance for complete tumor resection and re-expansion of the rest lung. We searched and reorganized relevant methods in terms of different emphasis on the pulmonary, compared and summarized their merits and drawbacks to provide a comprehensive depiction for surgeons to choose.

From a historical perspective, inflating gas into the lung was the method most likely to come to mind and was considered the first to reveal the segmental borderline in bronchiectasis. The positive pressure used by this primitive approach was discarded by subsequent methods to weaken the unexpected diffusion, but the expansion of the whole lung indeed impairs the view of surgeons. The inflation–deflation method put the inflation process after dissection of segmental hilar to reduce the interference on surgical view and soon became prevalent for its simplicity and economy. However, it may go ineffective in patients with abnormal pulmonary function. A selective jet injection is another way to highlight the target segment by gas inflation, but the requirement of a special facility and a high level of cooperation between surgeons and anesthesiologists limits its utilization. Staining of the segment through separated bronchus with safe dyes is developed based on the same principles and is being studied rapidly.

Intravenous drug administration is totally another different way that exploits disparity in blood supply and is developed in recent ten years. After the separation of the target bronchus and vessels, the indicator carried by blood flow distinguishes the rest part of the lung expeditiously, regardless of the patient’s pulmonary function. Yet, besides the demand for specialized cameras, the potential toxicity of dyes concerns surgeons. Also, a longer illuminating time for the operation is also required.

As a whole, the inflation–deflation method and intravenous ICG injection still served as the two main ways due to their respective irreplaceable advantages. Nevertheless, their drawbacks are in need of more investigation to overcome.

### Future

#### Simulation Based on Images Analysis

All of the above methods have high requirements for the accurate identification of segmental structures. Pre-operative three-dimension reconstruction based on computed tomography (CT) images is gradually becoming a useful implement (43) for surgeons to imagine the target segment in the brain. The technique of reconstruction was developing so rapidly that now is even applied to intraoperative guiding with video-assisted thoracoscopic lung surgery (VATS) by virtual simulation (44). Likewise, the intersegmental plane can also be simulated and facilitates the design of excision range and intra-operative recognition (45). Moreover, the intersegmental plane can be computed according to artery, bronchus, or vein, which may be valuable for the selection of resected structures (46).

It seems that computer technology can do much more than that. As the intersegmental plane depicted now is mainly on an inflated lung, we envisage that a deflated lung and its segmental borderline, which are closer to reality, can be simulated according to pulmonary volume or even by machine learning based on numerous images. A simulation of the lung after the inflation–deflation or other method is also available in the future. Furthermore, virtual reality on the basis of the data above brings more assistance to intraoperative management.

#### Safer Indicator

An indicator is essential for patients with poor lung functions, in which the inflation–deflation method may go ineffective. The ideal indicator should be nontoxic and can be observed in a relatively easy way. Given the toxicity of ICG that has been reported, safer visual indicators including vitamin B2 and heat discrepancy were applied and showed a promising outcome with satisfactory imaging. Self-colored dietary supplements and other nontoxic, fluorescent supplements are still under exploration.
